# Dental Implants: Modern Materials and Methods of Their Surface Modification

**DOI:** 10.3390/ma16237383

**Published:** 2023-11-27

**Authors:** Catherine Sotova, Oleg Yanushevich, Natella Kriheli, Sergey Grigoriev, Vladimir Evdokimov, Olga Kramar, Margarita Nozdrina, Nikita Peretyagin, Nika Undritsova, Egor Popelyshkin, Pavel Peretyagin

**Affiliations:** 1 Department of High-Efficiency Machining Technologies, Moscow State University of Technology “STANKIN”, Vadkovsky Lane 3a, 127055 Moscow, Russia; s.grigoriev@stankin.ru (S.G.); n.peretyagin@stankin.ru (N.P.); n.khokhlova@stankin.ru (N.U.); egorpav2635@yandex.ru (E.P.); p.peretyagin@stankin.ru (P.P.); 2 Scientific Department, A.I. Evdokimov Moscow State University of Medicine and Dentistry, Delegatskaya St., 20, p.1, 127473 Moscow, Russia; mail@msmsu.ru (O.Y.); krikheli_ni@msmsu.ru (N.K.); vvevdokimov@rambler.ru (V.E.); dr.ovkramar@gmail.com (O.K.); margo-rizhik@mail.ru (M.N.)

**Keywords:** dental implant, Ti and its alloys, stainless steels, Zr and its alloys, Ta and its alloys, ceramics, surface modification techniques

## Abstract

The development of dental implantology is based on the detailed study of the interaction of implants with the surrounding tissues and methods of osteogenesis stimulation around implants, which has been confirmed by the increasing number of scientific publications presenting the results of studies related to both the influence of the chemical composition of dental implant material as well as the method of its surface modification on the key operational characteristics of implants. The main materials for dental implant manufacturing are Ti and its alloys, stainless steels, Zr alloys (including ceramics based on ZrO_2_), and Ta and its alloys, as well as other materials (ceramics based on Al_2_O_3_, Si_3_N_4_, etc.). The review presents alloy systems recommended for use in clinical practice and describes their physical–mechanical and biochemical properties. However, when getting into the body, the implants are subjected to various kinds of mechanical influences, which are aggravated by the action of an aggressive biological environment (electrolyte with a lot of Cl^−^ and H^+^); it can lead to the loss of osteointegration and to the appearance of the symptoms of the general intoxication of the organism because of the metal ions released from the implant surface into the biological tissues of the organism. Since the osteointegration and biocompatibility of implants depend primarily on the properties of their surface layer (it is the implant surface that makes contact with the tissues of the body), the surface modification of dental implants plays an important role, and all methods of surface modification can be divided into mechanical, physical, chemical, and biochemical methods (according to the main effect on the surface). This review discusses several techniques for modifying dental implant surfaces and provides evidence for their usefulness.

## 1. Introduction

According to the World Health Organization (WHO), oral diseases are prevalent noncommunicable diseases that afflict almost half of the world’s population, i.e., around 45% or 3.5 billion individuals of all ages [1,2]. Complete tooth loss, or adentia, is steadily increasing with a peak occurrence in older age groups. Currently, there are over 350 million cases worldwide, resulting in a global prevalence rate of almost 6.82% [1]. In Russia, partial tooth loss is estimated to affect between 40% and 75% of the population, corresponding to a range of 58 million to 108 million people. And, the number of patients with such problems will increase as the average age of the population rises [3]. In addition to tooth loss caused by accidents (trauma), dental decay and gum diseases are key factors contributing to teeth loss. Untreated caries in permanent teeth affect 2.3 billion individuals, while over 514 million children suffer from untreated deciduous teeth caries, and more than 1 billion people, which represent 19% of the world’s population over 15 years of age, have periodontal disease [1].

Thus, the development of the dental implants market is driven by the high prevalence of oral diseases and the goal of improving people’s quality of life. The worldwide market for dental implants was estimated at $9.27 billion in 2022 and $10.09 billion in 2023, with a forecasted compound annual growth rate (CAGR) of 8.95% to hit $18.42 billion by 2030 [4]. The dental implants market is growing due to a rise in tooth loss cases, increasing demand for cosmetic dentistry, and advancements in dental implant technology [5].

**Figure 1:**
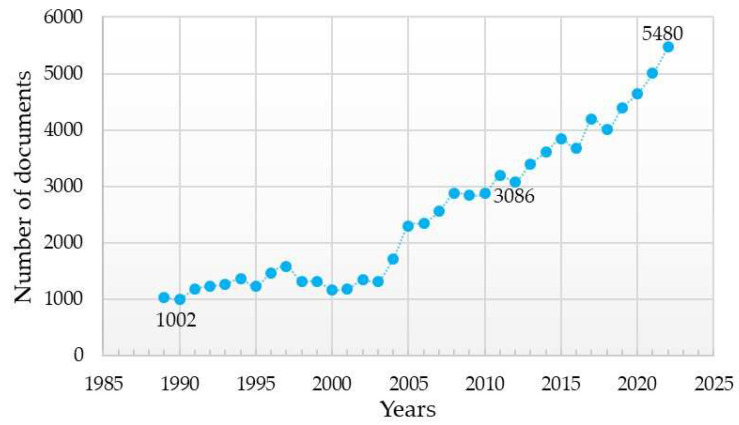
Analysis of search results for publications using the keywords “dental implant” from 1989, the year when the first patent for a titanium implant was registered, up to the present (according Scopus, ScienceDirect).

The advancement of dental implantology is based on a detailed study of the interaction of implants with surrounding tissues and methods of stimulating osteogenesis around implants. This has been demonstrated by the increasing number of scientific articles presenting the results of studies on the influence of the chemical composition of the dental implant material and the method of its surface modification on the key characteristics of dental implants (Figure 1). From 1989 (the year of the first patent registration for a titanium implant) to the present day, a search query with the keywords “dental implant” using bibliographic databases Scopus and ScienceDirect yielded 89,535 publications. Over the last decade (from 2012 to 2022), the number of publications has more than doubled (from 42,762 to 89,535).

**Figure 2:**
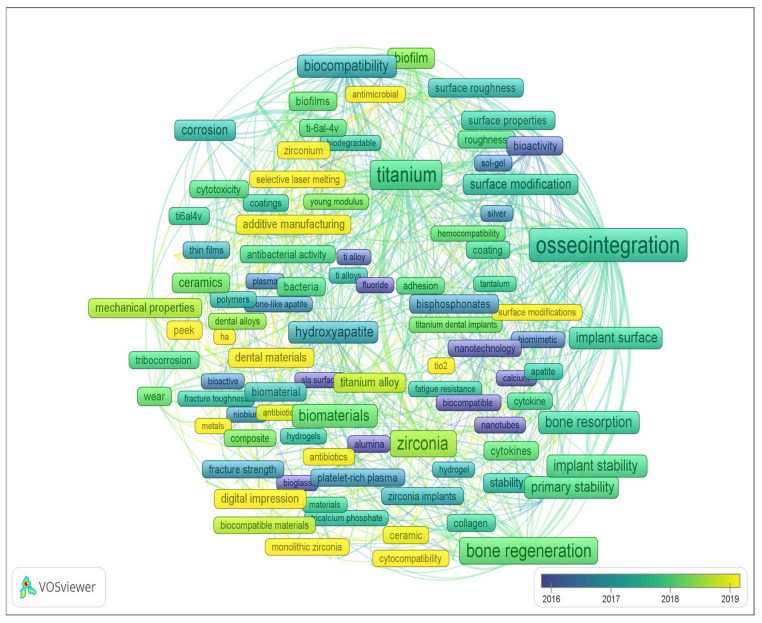
Overlay visualization of word cloud based on the keywords (dental implant) of publications from 2014 to present (according Scopus, ScienceDirect).

**Figure 3:**
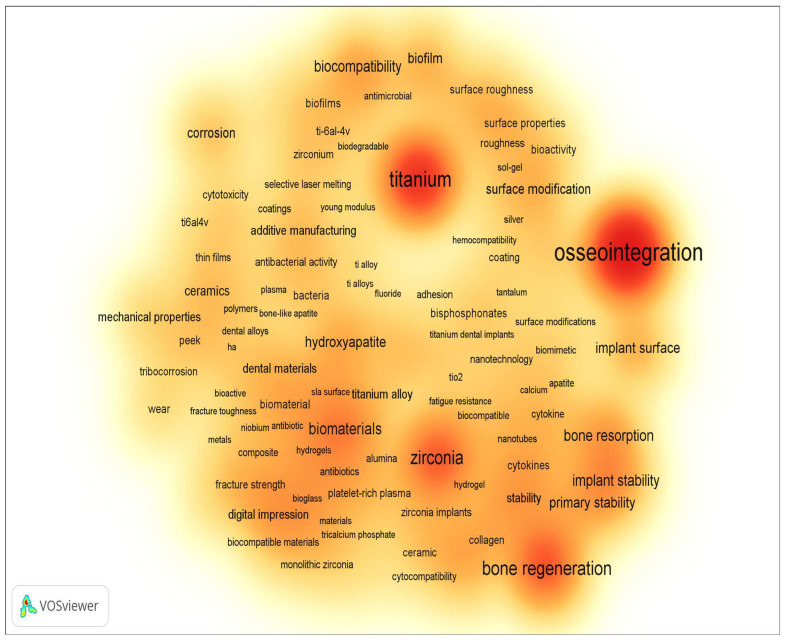
Density visualization of word cloud based on the keywords (dental implant) of publications from 2014 to present (according Scopus, ScienceDirect).

Figure 2 and Figure 3 display the word cloud generated by keywords from the previously mentioned sample, limited to the period from 2014 to the present, where keywords related only to implant material and surface modification methods and properties were selected. According to the results presented, the main requirements for implants are to provide and/or enhance the following properties (these have been the focus of scientific research on the improvement of dental implants):

- Osseointegration, or the fusion of the implant surface with the bone [6,7,8,9,10];
- Bone regeneration, the process by which the body builds new bone tissue [11,12,13,14,15];

- Antibacterial ability, the ability of the implant to inhibit or slow down bacterial growth [16,17,18,19,20];
- Biocompatibility, the ability of the material to be integrated into the body without clinical complications and to induce the required cellular or tissue response [9,21,22,23,24,25].

Based on their biocompatibility, materials for dental implants can be divided into biotolerant, bioinert, and bioactive [26]. Biotolerant materials, such as stainless steel and cobalt–chromium alloys, induce osteogenesis in the bone to respond to the irritating effect of the implant in the tissue contact zone. In this case, a layer of soft fibrous tissue separates the bone from the implant composed of these materials. The application of bioinert materials, including alumina, zirconia, titanium, its alloys, and tantalum, leads to the development of contact osteogenesis given favorable mechanical conditions. This means that these materials directly bond with the bone tissue. Bone integration occurs because the surface of such materials is chemically inert to the surrounding tissues and fluids. Bioactive materials such as calcium phosphate ceramics, glass, and glass ceramics cause connective osteogenesis—the direct chemical bonding of the implant with the surrounding bone—due to the presence of free calcium and phosphate on the surface and of their interaction.

In addition, a number of scientific studies have been devoted to the influence of the above-mentioned factors on other properties of implants, for example:

- Bone resorption—the destruction (resorption, degradation) of bone tissue under the action of osteoclasts [27,28,29];
- Biomechanical stability—the ability of the implant to be reliably fixed in the bone tissue without clinical mobility [30,31,32];
- Osteoconductivity—the ability of the implant to provide bone tissue formation and growth on its surface (a special case of osteointegration) [33,34,35];

- Osteoinductivity—the ability of the implant surface to induce differentiation in mesenchymal cells within the surrounding tissues, resulting in the formation of osteoblasts (a special case of osteointegration) [36,37,38];
- Osteogenesis—the ability of osteoblasts to form a new bone matrix (a special case of biocompatibility) [30,39,40,41];
- Osseoperception—the sensory feedback of sensorimotor, orofacial, and masticatory functions resulting from the direct transmission of forces and vibrations to bone tissue [42,43,44];
- Corrosion (or tribocorrosion) resistance—the ability of the implant not to be destroyed under the influence of the biological environment (saliva, blood, etc.) (reviewed in [45,46,47,48,49,50]).

**Figure 4:**
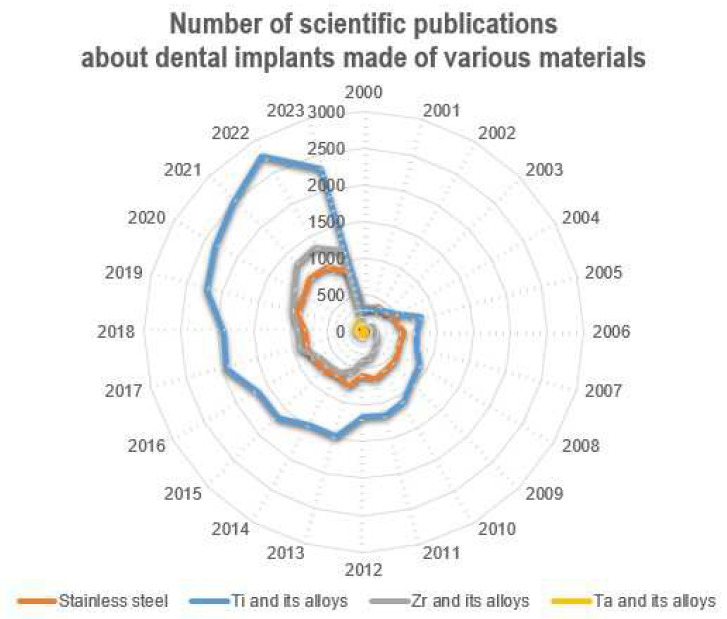
Number of scientific publications in the search results of publications for the keywords (dental implant*) from 2000 to the present (according to ScienceDirect).

Currently, according to numerous fundamental and applied studies, the main materials for dental implants are titanium and its alloys, stainless steels, zirconium alloys (including ceramics based on zirconium dioxide), and tantalum and its alloys, as well as other materials (ceramics based on aluminum oxide, silicon nitride, etc.). It should be noted that due to the lowest osseointegration and the presence of harmful impurities, the number of publications where the objects of research are implants made of stainless steels is significantly reduced (Figure 4). The great variety of dental implants based on chemical composition and surface layer modification technology indicates that the problem of choosing the optimal material for implant manufacturing has not been finally solved to date.

The purpose of this study was to analyze the different materials used in dental implantation at present (namely, for the production of dental implants representing the root of an artificial tooth), as well as to find ways to improve their performance properties by modifying the implant surface.

The material of the research comprised the literature data presented in scientific publications indexed in the bibliographic databases PubMed, ScienceDirect, and Scopus, using, as search terms, the keywords “dental implant*” with specifications on different groups of materials and methods of additional implant processing (surface modification techniques). The end date of the search was August 2023.

## 2. Dental Implant Materials

**Figure 5:**
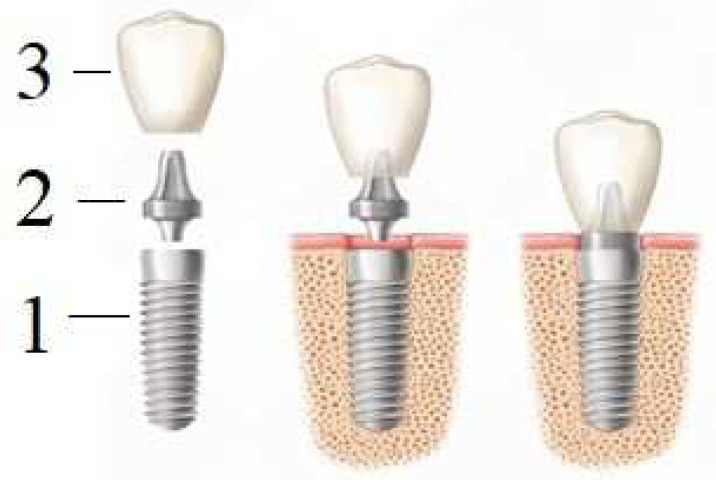
Dental prosthesis: 1—implant, 2—abutment, 3—crown.

An artificial tooth (denture) is a complex construction (Figure 5) consisting of an implant (1), abutment (2) and crown (3). The implant itself is completely immersed deep into the bone, and the crown is located above the gingiva; the abutments connect them with each other, except in the use of one-piece implants, in which the intraosseous part and the abutment are immediately connected into a monolithic element (one-piece type of implants). However, this variant is mainly used for full jaw restorations, i.e., basal implantation (in one-stage implantation protocols involving immediate loading with a prosthesis).

The implant material must meet several requirements [51]:

- To be strong enough to withstand chewing pressure (sometimes exceeding 100 kgf/cm^2^) and not to destroy the bone with its weight;
- To be well machinable at the manufacturing stage in order to retain its shape throughout its entire service life;
- To not be destroyed (not corroded) by the action of the biological environment (saliva, blood, etc.);
- To not show toxic, allergenic, and carcinogenic effects on the body;
- To not provoke an increase in galvanic currents when interacting with metal structures installed in the patient’s mouth.

**Figure 6:**
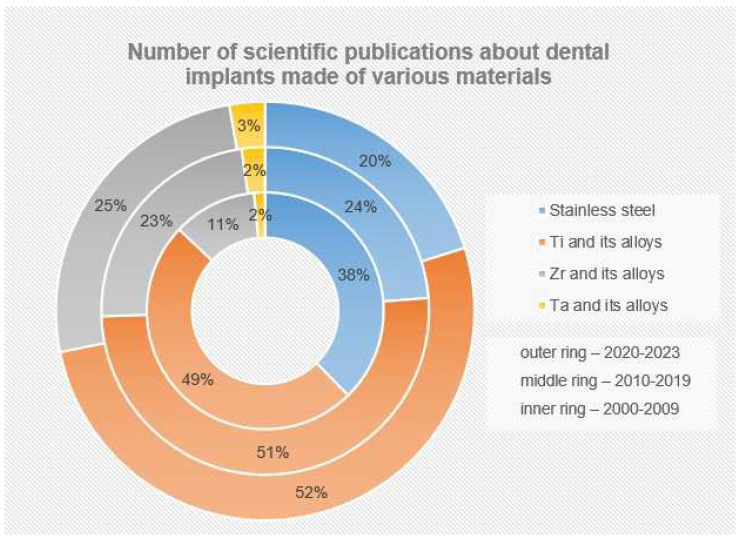
Number of scientific publications in the search results of publications for the keywords (dental implant*) from 2000 to the present (according to ScienceDirect). The number of publications (%) is presented relative to their total number for all groups of materials (SS+Ti+Zr+Ta).

The main materials for the production of dental implants, as mentioned before, are mainly metal alloys based on titanium, iron, and tantalum, as well as ceramics based on zirconium (Figure 6). At the same time, starting in the last twenty years, the number of studies in which the implants made of stainless steels are the objects of studies has considerably decreased (from 38% to 20%), and this has been connected with the presence of harmful impurities in the steel composition and the insufficient corrosion resistance and biocompatibility of these implants. At the same time, titanium and its alloys, during this period, have steadily occupied the leading positions (~50%), and today, they are the main materials for the production of commercialized dental implants.

### 2.1. Titanium and Its Alloys

The main materials for the production of dental implants are titanium and its alloys. For commercialized dental implants, the following grades of alloys are mainly used [52,53,54,55,56,57,58,59,60,61,62,63,64,65,66,67]:

- Grade 2 (cp-Ti) [53,54,55,56,57]—commercial pure titanium containing up to 0.3% iron;
- Grade 4 (cp-Ti) [57,58,59,60,61], which contains about 99% titanium and up to 0.5% iron;
- Grade 5 (Ti-6Al-4V alloy) [62,63,64,65,66,67], which is an alloy of titanium (88%) with aluminum (up to 6.75%) and vanadium (up to 4.5%);
- Roxolid^®^, which is a high-strength alloy of titanium (about 85%) with zirconium (~13–17%), specially developed for use in dental implantology [68,69,70,71].

The chemical and mechanical properties of these alloys meet the requirements for materials used in dental implantation. They are characterized by high strength at low density, high elasticity (five times higher than the elasticity of bone [26]), and bioinertness [72], but not all titanium alloys are used in dentistry. However, with all these positive properties of titanium and its alloys, they are characterized by low resistance to shear and wear, especially under friction conditions [26].

At the same time, dental implants should be designed with a high coefficient of friction and a low modulus of elasticity to avoid excessive stress on the bone [73]. The modulus of elasticity of solid titanium alloys is 100–115 GPa, while the values for different types of bone are 0.5–20 GPa. This mechanical mismatch is the reason why healthy bones are underloaded and the “stress shielding” effect occurs [74]. The “stress shielding” effect leads to the healthy bone resorption and loosening of the implant, as a result of which the implant functioning period decreases.

This is why studies on obtaining “new”-generation titanium alloys with mechanical properties approaching the properties of bone tissue are conducted widely enough. All of them are aimed at the volumetric alloying of titanium alloys with β-stabilizers (Nb, Ta, Zr, and Mo) to obtain a single-phase β-alloy structure since β-Ti alloy has a lower modulus of elasticity and higher strength than α-Ti alloys and (α+β)-Ti alloys [75]. In addition, the β-type titanium alloy has good technological properties (in particular, cold-pressure machinability), which reduces the cost of implant production [76].

Table 1 shows the compositions of titanium alloys, used for the production of dental implants, identified by the above search query.

Ti-6Al-4V alloy is 3.0–3.5 times stronger than commercially pure titanium and cheaper to produce [136], which makes it indispensable in the manufacture of thin, reliable implants with special compression threads that are placed in the dense basal regions of the jawbone [52]. However, vanadium and aluminum contained in the alloys have been shown [137,138] to have toxic effects on biological entities and can lead to long-term health problems, such as neurological diseases and Alzheimer’s disease [139,140,141,142], while aluminum and iron (although it is not a toxic element) lead to the formation of a connective tissue layer around the implant and to significant tissue contamination, which is a sign of the insufficient bioinertness of the metal. In addition, iron suppresses the growth of organic cultures. Also, the degree of tissue adhesion to the implants made of titanium alloys is somewhat worse than that to unalloyed titanium [143].

The introduction of Nb into the alloy composition, instead of toxic vanadium, improves the bioinertness of alloys and leads to a significant increase in the wear resistance of alloys of the Ti-Al-Nb system in comparison with Ti-Al-V alloys, with comparable values in terms of fatigue strength and the service lives of products. Thus, it was shown in [77] that the wear resistance of Ti-21Al-29Nb and Ti-15Al-33Nb alloys was more than three times higher than that of Ti-6Al-4V. At the same time, at low- and multi-cycle fatigue tests on samples from both alloys, the following were observed: the nucleation of surface cracks, furrows in the zone of propagation of a stable fatigue crack, and equiaxed dimples in the zone of propagation of a fast-fatigue crack. The Ti-15Al-33Nb alloy exhibited ductile fracture morphology whereas the Ti-21Al-29Nb alloy tended to exhibit a more brittle fracture morphology.

The introduction of yttrium-stabilized zirconium oxide into the alloy of the Ti-Al-Nb system increases the Vickers microhardness and biocorrosion resistance of the alloy and improves the corrosion resistance of the alloy [81].

In recent years, non-toxic titanium alloys with a lower modulus of elasticity and that are free of aluminum and vanadium, such as Ti-Nb, Ti-Zr, Ti-Ta, Ti-Fe, Ti-Mo, and others, have been used as alternatives to Ti-Al-based alloys (see Table 1). Among them, Ti-Nb-based alloys have a low elastic modulus, shape memory, and hyperelasticity, so these alloys are chosen as the base alloys for alloying studies by most biomedical materials researchers [53,82,83,84,85,86,87,88,89,90,91,92,93,94,95,96,97,98]. Thus, the elastic modulus of Ti-Nb alloys depends on the Nb content and has two minima: ~ 68 GPa at 15 wt.% Nb (the alloy has an (α+β)-structure) and 62 GPa at 42 wt.% Nb (the alloy has a β-structure) [82] (for comparison, the elastic modulus of Ti-6Al-4V alloy is ~ 115 GPa [94]).

Moreover, Nb is non-toxic and has no harmful effect on the human body, which can be a result of its ability to form a protective oxide film on the implant surface like titanium. According to a study [144], Nb demonstrated good compatibility in contact with cells, providing mitochondrial activity and cell growth. Moreover, Ti-Nb alloys release fewer metal ions into the surrounding tissues compared to Ti-Al-V, Ti-Ni, and Ti-Mo alloys [83,145]. Ti-Nb alloys also have good mechanical strength (e.g., for Ti-42Nb alloy, the tensile strength is 683.17 ± 16.67 MPa and the compressive strength is 1330.74 ± 53.45 MPa [82]) and hardness (microhardness increases with increasing Nb content in the alloy—276 HV_0.5_ for Ti-13Nb and 287 HV_0.5_ for Ti-28Nb [146]) because Nb dissolves in the Ti crystal lattice, forming a solid substitution solution, which leads to the hardening of the solid solution β [147].

While, in binary Ti-Zr alloys, zirconium has almost no effect on β-phase stabilization, in multicomponent Ti alloys containing Nb or Ta, Zr acts as an effective β-stabilizer [76]. At the same time, Ti-Nb-Zr (TNZ) alloys have high strength (the tensile strengths of the alloys are in the range of 704–839 MPa) while remaining at the level of Ti-Nb alloys in terms of their low modulus of elasticity (in the range of 62–65 GPa) [76]. In addition, the new series of β-TNZ alloys have excellent cytocompatibility [76,85]. According to [85], due to its good mechanical properties (E = 84.1 GPa), high corrosion resistance, and lack of cytotoxicity toward MC3T3 and NHDF cells, this Ti-13Nb-13Zr alloy can be successfully used in implants, including in bone tissue engineering and dental products.

The additional alloying of a TNZ alloy with tantalum (TNZT alloy) leads to an increase in the corrosion resistance of the alloy in the environment of biological fluids (e.g., saliva, biofilm, and fluoride) while maintaining (with a decreasing trend) a relatively low modulus of elasticity (E = 82 GPa) [88]. The excellent biological and corrosion performance results are achieved mainly due to the oxide film (TiO_2_, Nb_2_O_5_, ZrO_2_, or Ta_2_O_5_) spontaneously formed on Ti and its alloys when exposed to atmospheric air [88]. The introduction of silicon into the composition of the TNZT alloy makes it possible to further reduce the elastic modulus (up to 50 GPa) [90]. At the same time, the Ti-Nb-Zr-Ta-Si alloy has a significant compatibility with bone tissue in comparison with the cp-Ti alloy, which is widely used in dental implants. Thus, in in vitro tests, in comparison with cp-Ti, Ti-Nb-Zr-Ta-Si alloy showed better adhesion, proliferation, and alkaline phosphatase (ALP) activity and promoted the expression of osteocalcin (OCN) mRNA in MG63 cells [90].

The addition of Mo not only stabilizes the β-phase of alloys but also increases the strength and maintains the ductility of Ti-Nb alloys, while Ti-Nb-Mo alloys are not prone to riveting [75]. This leads to a decrease in the modulus of elasticity, which is lower the more Mo is present in the alloy composition. Thus, the reduced elastic modulus (Er measured via nanoindentation) decreases from 67.0 GPa for the Ti-26Nb-2Mo alloy to 54.5 GPa for the Ti-26Nb-8Mo alloy (with the lowest elastic modulus) [75]. In addition, the Ti-26Nb-8Mo alloy has good wear resistance and higher impact toughness than the cp-Ti alloy.

Despite the biocompatible composition, improved corrosion resistance, and low modulus of elasticity of complex-alloyed titanium alloys, serious aspects limiting the effectiveness of titanium alloys for biomedical applications are low tribocorrosion resistance in biological body environments and an inability to prevent bacterial infection in the early stages of recovery [148,149]. The latter can be addressed by alloying with antibacterial components such as Cu, Zn, Ag, and Ga [150].

In one study, although the addition of alloying elements Ga and/or Cu to the Ti-Nb matrix resulted in a slight increase in the elastic modulus to 73–78 GPa (for the base alloy Ti-45Nb, E = 64 GPa), the elastic modulus was still lower than that of the alloys used in clinical practice [93]. Among β-Ti-Nb-Ga alloys, increased wear resistance was noted for the Ti-45Nb-8Ga alloy [94], which is associated with increased microhardness (it increased by 32–44% with the addition of Ga and/or Cu, which the authors [93] attributed to the hardening of solid solutions). Also, the addition of Ga to Ti-45Nb leads to both improved corrosion resistance under mechanical loading as well as the increased strength of the alloy [94].

Another group of alloys used for the fabrication of dental implants, which have been clinically approved and already successfully commercialized, comprises Ti-Zr alloys [68,69,70]. For example, Ti-Zr alloys can compete in the fabrication of small-diameter dental implants. In these cases, cp-Ti is more prone to fracture (due to its relatively low compressive strength), and the use of Ti-Al-V alloy is undesirable due to the toxicity associated with the release of Al and V.

For binary Ti-Zr alloys (as mentioned earlier), Zr is a neutral component with respect to titanium and easily dissolves in both α- and β-solid substitution solutions of unlimited solubility of Ti(Zr) [151] (while strengthening it due to distortion of the crystal lattice of titanium when its atoms are replaced by zirconium atoms [152,153]) since it undergoes a similar allotropic transformation at a close phase transition temperature [154].

Ti-Zr alloys have a predominantly α-crystalline structure, which predetermines the enhanced mechanical properties and excellent electrochemical properties of these alloys [150,153]. Thus, the addition of 5 and 10 wt.% Zr to Ti allowed more than doubling the microhardness of the alloy (HV 416–434) compared to the microhardness of pure titanium cp-Ti (HV ~188), surpassing even the hardness of the Ti-6Al-4V alloy (HV 354) [155]. The tensile strength of binary Ti-Zr alloys also increases with increasing zirconium concentration in the alloy. In [101], it was shown that the tensile strength of Ti-15Zr alloy exceeds that of cp-Ti by about 10–15% (950 MPa vs. 860 MPa, approximately), which the authors attributed, first, to the hardening of the solid solution due to alloying, second, to the refinement of its grain size (1–2 μm in Ti-15Zr as opposed to 20–30 μm in cp-Ti), and third, to the riveting obtained during sample fabrication (strain hardening). However, Ti-Zr alloys have an increased modulus of elasticity, and the modulus of elasticity increases with increasing zirconium concentration in the alloy (for Ti-5Zr, Ti-10Zr, and Ti-15Zr alloys the values were ~86, ~95, and ~110 GPa, respectively [152]), but for Ti-Zr alloy with a Zr concentration of up to 10 wt.%, the modulus of elasticity was significantly lower than for the widely used Ti-6Al-4V alloy, which had a higher elastic modulus (~115 GPa [94]). At the same time, the minimum modulus of elasticity has an alloy containing about 7.5 wt.% Zr [152].

Also, Ti-Zr alloys demonstrate increased corrosion resistance in comparison with traditional titanium alloys, which can be associated with the formation of passivating ZrO_2_ film on the implant surface. Thus, Zr for Ti is an anodic alloying component, which directly reduces its anodic activity [156], i.e., ZrO_2_ is a more stable oxide than TiO_2_. And, although Ti^4+^ ions are more mobile than Zr^4+^ ions, which leads to a significant reduction in the amount of ZrO_2_ oxide in the outer surface layer, the amount of the latter is proportional to the concentration of Zr in the alloy [155]. Higher corrosion protection for Ti-Zr alloys may also be facilitated by their increased hardness (and therefore wear resistance), which may prevent damage to the passivation film by mechanical stresses and ensure long-term rehabilitation success by reducing both the likelihood of corrosion in physiologic environments and the likelihood of impaired osseointegration [157]. In addition, it was shown in a study [155] that higher Zr concentrations resulted in enhanced albumin adsorption (albumin adsorption values for cp-Ti, Ti-6Al-4V, Ti-5Zr, and Ti-10Zr were 600, 650, 650, 750 mg/mL, respectively), suggesting, according to the authors, that there was no negative effect on initial cell adhesion.

Due to the sensitivity of mechanical properties to the structure of binary Ti-Zr alloys, it is possible to reduce the modulus of elasticity by applying strengthening heat treatment (accelerated cooling after heating above the β→α transformation temperature) or strain hardening, which will change the distance between atoms, and this, in turn, can lead to a change in the bonding force between atoms and, as a result, the modulus of elasticity [152,158]. In addition, the use of additive technologies to create porous structures can also contribute to a decrease in the elastic modulus while maintaining the relatively high strength of the alloys. For this connection, additional studies are needed to reveal the influence of technologies for producing products from binary Ti-Zr alloys on their mechanical properties.

The additional alloying of Ti-Zr alloys with β-stabilizers (Nb, Mo and/or Ta) leads to a decrease in the elastic modulus [104,105,155] since the introduction of these components promotes the formation of β-phase in the structure, characterized by the lowest elastic modulus among all phases of titanium alloys [159]. Nb reduces the hardness of Ti-Zr alloy to a value close to the value of cp-Ti (~ 200 HV) [155], which is also associated with the appearance of a softer β-phase in the structure. The influence of zirconium concentration on the hardness of ternary Ti-Nb-Zr alloys is similar to that of binary Ti-Zr alloys; a study [155] showed the higher hardness of Ti-Nb-Zr alloys with higher Zr concentrations (in the range of zirconium concentrations of up to 10 wt.%).

However, as shown by studies, in complex alloys such as Ti-Zr-Nb-Mo, increasing the concentration of Zr from 34 to 40% (by replacing it with niobium while keeping the content of titanium and molybdenum constant) leads to a decrease in hardness (243 ± 4, 2 HV, 238 ± 2.74 HV, and 237 ± 3.07 HV for Ti-34Zr-17Nb-1Mo, Ti-37Zr-14Nb-1Mo, and Ti-40Zr-11Nb-1Mo alloys, respectively) and alloy elastic modulus (75, 72, and 69.5 GPa for Ti-34Zr-17Nb-1Mo, Ti-37Zr-14Nb-1Mo, and Ti-40Zr-11Nb-1Mo alloys, respectively) [105] (in contrast to alloys with low zirconium content, i.e., up to 15 wt.%, for which hardness and elastic modulus increase with an increasing Zr concentration in the alloy [152]).

Biosafety evaluation tests (in accordance with the ISO 10993 series, [160]) of Ti-Zr alloyed with Nb and Ta [104] did not reveal any adverse (negative) effects either during extraction simulating normal use or under conditions of exaggerated extraction, which the authors attributed to the small number of released Ti ions. Thus, the concentrations of Ti in 0.9% NaCl + HCl solution when the samples from Ti-15Zr-4Nb, Ti-15Zr-4Nb-1Ta, and Ti-15Zr-4Nb-4Ta alloys were immersed in it were 0.4, 0.27, and 0.32 μg/mL, respectively. The authors of the study [104] substantiated such results via the presence of a passivating TiO_2_ film formed on the Ti alloy surface and containing Nb_2_O_5-_, ZrO_2-_, and Ta_2_O_5-_ oxide films, which inhibited the yield of metal ions.

Another study [106] showed the prospects of the biomedical application of Ti-Zr alloys additionally alloyed with Fe. The Ti-6Zr-xFe alloy has an (α+β)-structure. With an increasing Fe content, from 4 to 7 wt.%, the volume fraction of α-phase decreases, the fraction of β-phase increases, and the grain size of the alloy as a whole decreases. This leads to an increase in microhardness (264–348 NV), the tensile strength (748–994 MPa) of cast Ti-6Zr-xFe alloys due to the hardening of the solid solution (due to iron alloying) and formation of ω-phase, and elastic modulus (90–94 GPa). At the same time, the alloys Ti-6Zr-4Fe and Ti-6Zr-5Fe (with the lowest Fe content) showed better corrosion resistance.

In each of the binary titanium alloys Ti-Ta and Ti-Mo, alloying with tantalum or molybdenum leads to higher strength and lower modulus of elasticity in the alloy compared to cp-Ti (similar to the effect of niobium) since Ta and Mo are also β-stabilizers. Thus, in a study, Ti-40Ta and Ti-50Ta alloys had higher tensile strength values compared to Ti-6Al-4V (786, 724, and 689 MPa, respectively), which were 14% and 8.9% higher than that of Ti-6Al-4V material [161], and the Ti-40Ta alloy with a biomimetic lamellar structure (obtained using sequential spark plasma sintering at 1200 °C followed by hot rolling at a strain rate of 60% and annealing) had a suitable combination of strength (tensile strength of 980 MPa, which was much higher than cp-Ti and comparable to Ti-6Al-4V) and low modulus of elasticity (80.4 GPa) [108]. It is worth noting that the strength and modulus of elasticity of Ti-Ta alloys are very sensitive to the Ta content; the dependence of these properties on the tantalum content in the binary Ti-Ta alloy has a complex character. Thus, the elastic modulus of the alloy first decreases almost linearly with an increasing Ta content and reaches a local minimum of 69 GPa at 30% Ta, then gradually increases and reaches 88 GPa at 50% Ta, and then gradually decreases and reaches a second local minimum of 67 GPa at 70% Ta. A further increase in Ta content leads to an increase in the elastic modulus, approaching the modulus of pure Ta [162]. The opposite dependence is observed for the tensile strength: at first, with an increasing Ta content, the strength increases, and the tensile strength reaches the first local maximum (595 MPa) at 30% Ta, then the strength slightly decreases to 530 MPa at 50% Ta. After that, it increases again and reaches the peak value of 690 MPa at 60% Ta; a further increase in Ta content slightly decreases the strength of the alloys [162]. The authors of [162] attribute these changes to the influence of tantalum concentration on the structure of binary Ti-Ta alloys (in their study, the alloys had a hexagonal (α’) martensitic structure with a Ta content of up to 20%, orthorhombic (α”) martensitic structure at a Ta content from 30 to 50%, (α” +β)-structure in the alloys with 60% Ta, and single-phase metastable β structure at a Ta content of more than 60%). In addition to tantalum concentration, the structure and, consequently, the properties of binary Ti-Ta alloys (by changing the phase composition and their ratio) are affected by the hardening heat treatment and plastic deformation (as confirmed by studies [162]).

In addition, Ti-Ta alloys have high biocompatibility and corrosion resistance. Thus, the results of a study [111] have confirmed that the corrosion resistance of Ti-Ta alloys with tantalum contents of 30, 40, 50 and 60 wt.% are not inferior to that of the Ti-6Al-7Nb alloy, and in fluorinated acidified saliva, even exceed it, which can be explained by the formation of Ta_2_O_5_ oxide film on the surface of a Ti-Ta alloy. At the same time, the authors of another study [112] state that a minimum of 40 wt.% Ta is recommended to achieve excellent corrosion properties in Ti-Ta alloys.

Tantalum also exhibits antibacterial activity against various pathogens such as Staphylococcus aureus and Escherichia coli [163,164], so further studies on the antibacterial activity of Ti-Ta alloys are needed.

**Figure 7:**
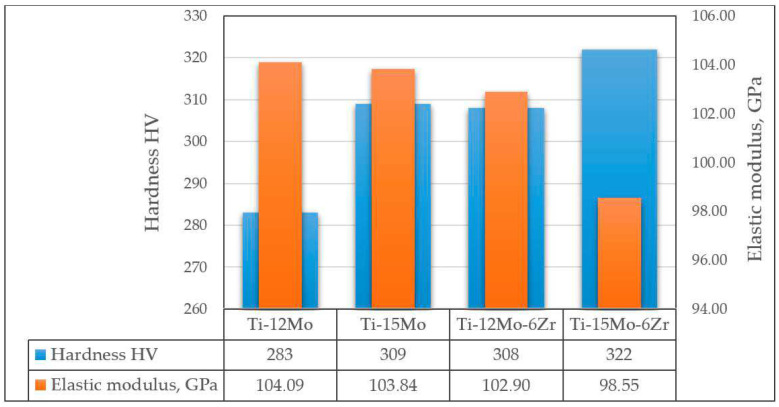
Hardness and elastic modulus of Ti-Mo and Ti-Mo-Zr alloys according to data [123].

As mentioned above, molybdenum acts as a strong β-stabilizer, which reduces the elastic modulus and increases the corrosion resistance of binary Ti-Mo alloys. Thus, Ti-Mo alloys with Mo content of up to 12% have lower elastic moduli than cp-Ti (138.56 GPa) [165]. However, the character of the influence of Mo concentration (in the concentration range from 3.2 to 12 at.%) on the elastic modulus of the alloy is identical to that of tantalum: the first local minimum corresponds to the Ti-3.2Mo alloy (83.8 GPa), the local maximum to Ti-6Mo (112.092 GPa), and the second local minimum to Ti-8Mo (82.98 GPa). It is worth noting that a significant increase in the elastic modulus between Ti-4.5Mo and Ti-6Mo alloys is observed when the Mo content increases up to 6 at.%, then its value falls sharply when the Mo content increases from 6 to 8 at.%, and when the Mo content changes from 8 to 12 at.%, only a slow increase is observed [165]. A further increase in the amount of molybdenum in binary alloys, as well as their additional alloying with zirconium, leads to an increase in the amount of β-phase in the alloy structure, which contributes to an even greater decrease in the elastic modulus while maintaining the increased strength of the alloy. In [123], it was shown that the ratios of α/β-phase (in %) for Ti-12Mo, Ti-15Mo, Ti-12Mo-6Zr, and Ti-15Mo-6Zr alloys were 53.7/46.3, 50.61/49.39, 40.03/59.97, and 16.26/83.74, respectively. This also led to an increase in the hardness of the alloys and the elastic modulus of the alloys (Figure 7).

The introduction of Fe into the ternary alloy of the Ti-Mo-Zr system reduces the elastic modulus of the alloy (from 105 GPa to 93 GPa with the addition of Fe from 1 to 3% [125]), while Ti-Mo-Zr-Fe alloys (containing from 1 to 4% Fe) are pseudo-β-alloys, i.e., the structure of the alloys contains up to 99% of the β-phase, the grain size of which decreases with increasing iron content [125].

Ti-Cu alloys are promising antibacterial biomaterials that have the potential to be tools against peri-implantitis and antibiotic resistance [117]. It was found that by varying the Cu concentration (1 to 10 wt.%) and aging temperature, the antibacterial properties of Ti-Cu alloys could be controlled. Thus, the best antibacterial properties were observed in the alloy with the maximum Cu content, Ti-10Cu, which was aged at 400 °C for 6 h [117], which may have been due to the high content of Ti_2_Cu in the aged alloy and, as a consequence, a higher rate of release of Cu ions. It is worth noting that this alloy was the only material that showed an antibacterial effect after two hours of testing, while after six hours, bacteria were destroyed in all alloys with a Cu content of more than 5 wt.%.

Ti-Mg alloys have a low modulus of elasticity while maintaining the high specific strength and corrosion resistance inherent in cp-Ti (while the corrosion resistance and compressive modulus of Ti-Mg alloys decrease with increasing amounts of Mg) [126,127,128]. Moreover, Mg can dissolve in the modeled body fluids and contribute to the formation of a calcium phosphate layer; after the degradation of Mg, it is possible to obtain a porous titanium framework that promotes bone sprouting into the implant volume, i.e., the osseointegration and bioactivity of the implant are increased [128].

The introduction of Sr slows down the degradation rate of Ti-Mg alloys and increases their biocompatibility and bioactivity [130]. Sr promotes bone tissue regeneration and osteointegration at the interface (bone–implant), stimulates protein amalgamation, prevents osteoporosis, and suppresses bone resorption.

The alloys of the Ti-Mg-Sr system showed high mechanical properties in comparison with traditional alloys (cp-Ti, Ti-Al-V). Thus, the Ti-10Mg-20Sr alloy showed a low elastic modulus of 36 ± 7 GPa (measured by nanoindentation) with a hardness of 1.8 ± 0.8 GPa [130].

The addition of calcium to both titanium as well as noble metals (in particular, Pd, Pt) leads to an increase in the corrosion resistance of the binary alloys Ti-Ca, Ti-Pt, and Ti-Pd in acidic fluoride aqueous solutions (e.g., HF + NaF) adapted to different pH values (from 4.7 to 3.4) [133]. This was confirmed in a study [134], where it was shown that titanium alloys Ti-0.2Pd and Ti-0.3Mo-0.8Ni exhibited higher corrosion resistance than cp-Ti at fluoride concentrations below 0.002 M due to the accelerating effect of Pd and Ni on the cathodic process and the inhibiting effect of Mo on the anodic process.

In addition, the introduction of calcium and palladium into the composition of a titanium alloy contributes to the improvement of the osteointegration of the alloy. Thus, after 60 days of exposure to immersion in SBF solution with the addition of bovine serum albumin (0.8 g/l), precipitated CaP compounds (with Ca:P ratios from 1:0.7 to 1:4.4) were found on the surfaces of Ti-0.2Pd alloy samples [135].

Thus, alloying titanium alloys with β-stabilizers (Nb, Mo, Zr (for triple and more alloys), Fe, etc.) leads to a significant decrease in the elastic modulus and increase in the strength of alloys in comparison with traditional commercially available alloys (cp-Ti, Ti-6Al-4V), but it is not enough (the minimum elastic modulus of titanium alloys is 40–50 GPa, and that of bone is 4–30 GPa). The further reduction of Young’s modulus is possible by creating porous structures with pore sizes of 100–500 μm for better ingrowth of new bone tissue [127,166]. The prospectivity of this has been supported by some studies. For example, it has been shown [127] that porous Ti fabricated using the high-pressure solid-state sintering (HPSSS) method has a high strength (~115 MPa), relatively low Young’s modulus (~18 GPa), excellent ductility, and suitable pore sizes (50–300 μm), which make it attractive for load-bearing implant applications. Meanwhile, the strength of porous titanium can also be improved by alloying with Mo and Nb. Thus, porous alloy Ti-12.5Mo, with a porosity of 40–45%, and alloy Ti-25Nb, with a porosity of 39–48%, sintered at 1050 °C for 2 h, have excellent properties close to the properties of human cortical bone (Young’s modulus is in the range of 5–18 GPa, with compressive strength in the range of 141–286 MPa), which correspond to the properties of bone [167]. In addition, in the study, the compressive strength and Young’s modulus increased linearly with the decreasing porosity of the samples.

As our study has shown, titanium and its alloys remain some of the promising materials for dental implants with excellent biocompatibility and osseointegration. And, their development follows the way of making the mechanical properties of titanium alloys approach the properties of bone tissue (due to alloying and the creation of meta-materials with porous 3D structures), increasing tribocorrosion properties (in particular, in acidic fluoride media, where titanium has low corrosion resistance), and giving the alloys antibacterial and bone tissue regenerating properties.

### 2.2. Zirconium and Its Alloys

In modern practice zirconium, and its alloys are used for dental implant manufacturing [168,169,170,171,172,173]. Comparing the values of the electrode potentials of titanium (-1.63 mV) and zirconium (-1.4 mV), it can be assumed that implants based on zirconium alloy are more preferable, which is due to the negative influence of the negative potential of the implantation material surface on the surrounding tissues [174]. The higher the negative value of the standard electrode potential of a metal is, the greater its solubility and reactivity will be [175].

The conducted analysis of the results of the above-mentioned search query has shown that two groups of zirconium-based alloys are used for dental implant manufacturing:

- Metal alloys of the Zr-Nb [176,177,178], Zr-Ti [153,179,180,181,182], Zr-Ti-Mo [168], Zr-Ti-Nb [183,184], Zr-Ti-Ag [179], Zr-Ta, and Zr-Ta-Ti [9] systems;
- Ceramic materials based on zirconium dioxide:
  ◦ ZrO_2_ [185,186,187,188,189,190];
  ◦ ZrO_2_ stabilized with cerium (Ce-TZP) [191,192];
  ◦ ZrO_2_ stabilized with yttrium (Y-TZP or YSZ) [193,194,195,196];
  ◦ Ce-TZP/Al_2_O_3_ nanocomposite, which is a Ce-stabilized ZrO_2_-based composite material reinforced with Al_2_O_3_ crystals or fibers (Al-TZP) [197,198,199,200].

Zirconium is a rather soft gray metal, but it has a lower modulus of elasticity (92 GPa), better corrosion resistance than Ti [201], and good biocompatibility and osteoinductivity [202]. Zr, similar to titanium, is allotropic. Therefore, metal alloys based on it (in particular, Zr-Nb) belong to the group of alloys with solid-solution hardening and differ from intermetallic alloys, i.e., those inclined to magnetization, which include titanium, based on the high characteristics of fatigue endurance that do not depend much on the metal structure [174].

In [178], the microstructure and mechanical properties of cast Zr-(0-24)Nb alloys were investigated. It was found that when Nb was introduced in small amounts (up to 6 wt.%), Zr-Nb alloys consisting mainly of α’-phase (containing less than 6 wt.% Nb) showed the highest strength (786–881 MPa), moderate ductility (6.5–3.7%), and relatively high Young’s modulus (74 GPa) while maintaining low magnetic susceptibility. A further increase in niobium content led to a decrease in alloy strength (tensile strength of Zr-22Nb alloy was 605 MPa), which was associated with both the appearance of ω-phase as well as an increase in the amount of a less strong β-phase. It is worth noting that Young’s modulus of Zr-Nb alloys at a niobium concentration of up to 24% (over the whole investigated range) was lower than the elastic modulus of the titanium alloy Ti-6Al-4V, with the minimum value of Young’s modulus (48.4 GPa) being associated with alloy Zr-20Nb. Although the strength of this alloy was inferior to that of the Ti-6Al-4V alloy (687 MPa vs. 994 MPa), its value exceeded that of bone strength, and the magnetic susceptibility of the Zr-20Nb alloy was half that of the Ti-6Al-4V alloys, making it a preferred implant material for patients requiring MRI studies [178].

As mentioned above, zirconium and titanium have close atomic sizes and allotropic transformation temperatures, which allows these elements to form solid solutions of unlimited solubility. The properties of binary alloys Ti-xZr (where x is up to 90 at.%) have been discussed in detail above. Let us only add that metallic zirconium has excellent corrosion resistance in acid or alkali solutions but is subject to localized corrosion in media containing chloride or fluoride. The introduction of titanium into the alloy composition reduces the local corrosion of Zr-Ti alloys (while increasing the titanium content contributes to a decrease in the corrosion rates of the alloys), but their resistance to pitting corrosion is still inferior to that of cp-Ti [179]. Zr-20Ti and Zr-40Ti alloys exhibit good mechanical properties (the ultimate strength values are 1630 MPa and 1884 Mpa, and the microhardness values are 330 HV and 340 HV, respectively) and good machinability in cold deformation (alloy deformations at fracture are 25.2% and 26.8%, respectively) [179].

Binary Zr-Ti alloys have a predominantly α-crystalline structure, and the introduction of molybdenum, which is a β-stabilizer, leads to a change in the alloy structure. With the gradual addition of Mo from 0 to 12.5 wt.%, the phase composition of the alloys changes as α → α + β → β, with the amount of β-phase increasing with the increasing Mo content [168]. The properties of Zr-Ti-Mo alloys fully meet the requirement of aligning with the mechanical properties of biomedical dental implants. Thus, the alloys Zr-25Ti-xMo (where x is from 0 to 12.5 wt.%) have a reduced compressive strength in the range of 1154.4 to 1310.8 MPa, yield strength of 673.2 to 996.0 MPa, and Young’s modulus of 17.74 to 24.44 GPa, and the alloy Zr-25Ti-7.5Mo shows the best wear and corrosion resistance [168].

The introduction of Nb into the composition of Zr-Ti alloys also leads to the change of its structure to the β-type. Thus, the microstructure of the cast Zr-19Ti-21,4Nb alloy is represented by β-grains of 150–200 μm in size. The mechanical properties of this alloy in the deformed state correspond to the properties of the Ti-6Al-4V alloy (the tensile strength of the Zr-19Ti-21,4Nb alloy was 850–900 MPa, and the deformation of the alloy at fracture, characterizing plasticity, was 10.5%), but the modulus of elasticity is much closer to that of natural bone (37.6 GPa) [183]. The experiment with cell cultivation demonstrated the adequate adhesion of osteoblasts on the first day to both sr-Ti and Zr-Ti-Nb alloys, with subsequent proliferation on days 3 and 7. These data indicate the biocompatibility of Zr-19Ti-21,4Nb alloy and the absence of toxicity to cells [183].

The introduction of silver (6%) into Zr-Ti alloys leads to increased resistance to electrochemical corrosion (in particular, in artificial saliva containing fluorine). Moreover, the effect of Ag is stronger the more titanium is in the alloy composition since the joint action of titanium and silver promotes the formation of a thick, compact, and stable passive film [179]. At the same time, silver slightly changes the mechanical properties of Zr-xTi alloys. Thus, in a study, the introduction of 6% Ag increased the hardness of the Zr-20Ti alloy from HV 329 to HV 338, and the hardness of the Zr-40Ti alloy increased from HV 340 to HV 349 [179].

The Zr-30Ta and Zr-25Ta-5Ti alloys also showed improved biocompatibility and osteogenic activity in comparison with cp-Ti due to, among other things, favorable surface properties (increased surface hydrophilicity and roughness) [9]. Thus, the Zr-25Ta-5Ti alloy had the smallest contact angle (~30.6°) and the largest roughness value (Ra = 34.8 nm) while, for the Zr-30Ta alloy, the values of the contact angle of wettability and roughness were 46.6° and 20.7 nm, respectively (for comparison, these characteristics for cp-Ti were 52° and 13.1 nm). The differences between the samples were statistically significant (*p* < 0.01). In addition, the Zr-30Ta and Zr-25Ta-5Ti alloys also induced the balanced expression of M1 and M2 macrophage phenotypes during the first 24 h. A Zr-25Ta-5Ti sample adsorbed three times more protein ((147.4± 13.26) μg) compared to Zr-30Ta ((32.1 ± 4.57) μg) and CP-Ti ((32.4 ± 3.7) μg) samples.

Unlike metallic zirconia, ZrO_2_, zirconium dioxide, is an ultra-hard material; at the same time, it has a white color similar to that of the natural tooth root, which is favorable for restorations in the smile area [186], and it is biocompatible, does not provoke allergies, and is one of the most durable in service [174]. The zirconia surface is poorly adhered to by bacteria (40% less adhesion than metals) [203,204], and ceramic materials are less prone to plaque accumulation than metal substrates [200].

Zirconium dioxide ceramics have higher strength (the bending strength of zirconium ceramics varies from 500 to 1200 MPa [205,206,207]) and crack resistance (not less than 15 MPa-m^1/2^ [206]); high acid-, corrosion-, and wear resistance due to high hardness (Vickers hardness of not less than 10 GPa [206]) and heat resistance; and biocompatibility in comparison with alumina analogs [174,208]. This material is characterized by an extremely low coefficient of friction with metals and the possibility of obtaining very high surface cleanliness [174]. However, compared to titanium alloys, zirconium dioxide has an increased modulus of elasticity [208] (100–200 GPa [206] vs. 30–115 GPa [75,76,82,90,94,209]), comparable to the modulus of elasticity of stainless steel (210 GPa [209]). In addition, ZrO_2_ has a high value of compressive strength (more than 2000 MPa [210]), significantly exceeding the compressive strength of titanium alloys (~400–1000 MPa [93]). It should be noted that the strength of ceramics depends on porosity and grain size, which, in turn, are influenced by the sintering technology and the addition of various modifiers to the ceramic composition, and the strength of titanium alloys depends on alloying additives and alloy manufacturing technology. Thus, [25] has shown that the compressive strengths of ZrO_2_ ceramics and Ti-6Al-4V alloy are practically equal (1525 MPa and 1565 MPa, respectively).

The introduction of stabilizing oxides of ceramics cerium (CeO_2_), yttrium (Y_2_O_3_), and aluminum (Al_2_O_3_), which form a solid solution with ZrO_2_, prevents spontaneous tetragonal–monoclinic transformation from occurring during the cooling of ceramics from the sintering temperature and contributes to grain refinement and leads to an increase in the bending strength of ceramics and ductility (it is known that when the grain size is reduced to values of the order of 1–5 microns, there is a decrease in porosity and an increase in strength and the amount of tetragonal zirconium dioxide [206]), which make it possible to withstand high torsional moments during implant placement. At the same time, the high compressive strength values of the ceramics are maintained.

Zirconium dioxide is also bioinert to other materials in the oral cavity and is particularly suitable for patients who are allergic or intolerant to metals [211]. The clinical use of zirconium oxide implants is practically free of peri-implantitis, and gingival epithelial tissues are able to firmly and reliably attach to their surfaces [212].

It was found that the osseointegration processes of implants made of ZrO_2_ and Ti are similar. Thus, the degree of contact of the implant with the surrounding bone after 4 weeks of functional loading (*p* = 0.505) for ZrO_2_ was 76.76%, and it was 77.25% for titanium, and the average total bone density after 4 weeks was 57.51% around zirconium implants and 60.89% around titanium implants (*p* = 0.223) [187]. A greater number of formed and shaped bone areas were found around the perimeter of bone contact areas with zirconium implants compared to bone contact areas with titanium implants, where erosion lacunae were more numerous [213].

It was also found that the stabilization of ZrO_2_ with cerium oxide leads to the increased bioactivity of ceramics (Ce-TZP nanocrystals directly bind to hydroxyapatite crystals deposited by osteoblastic cells without any preliminary chemical treatment of the substrate surface) [191]. Ce-TZP has extremely high values of fracture toughness (up to 30 MPa-m^1/2^) [206], but it is possible to increase the plasticity of Ce-TZP zirconia ceramics only by introducing no more than 10 mol% [192]. However, from an aesthetic point of view, Ce-TZP ceramics have a yellowish color, which limits the material’s use as a restoration for frontal teeth [214].

ZrO_2_ stabilized by yttrium (yttrium oxide) also has high values in terms of strength (up to 1300 MPa), Young’s modulus (~ 205 GPa), and hardness (10 GPa) [206]. At the same time, an increase in the toughness of ceramics is possible at a content of more than 3 mol% Y_2_O_3_. The results of a study on Y-TZP ceramics biocompatibility testify to the increased adhesion, viability, and proliferation of osteoblasts and gingival fibroblasts on zirconium surfaces in comparison with titanium ones, which the authors [195] attributed to the increased wettability of the zirconium implant surface. However, one of the disadvantages of Y-TZP is degradation during low-temperature aging; exposure to moisture for a long period of time, even at low temperatures in the mouth, can lead to material cracking and catastrophic implant failure [200,215].

Ce-TZP/Al_2_O_3_ nanocomposite is a Ce-stabilized ZrO_2_-based composite material reinforced with Al_2_O_3_ crystals of several hundred nanometers in size. Ce-TZP/Al_2_O_3_, containing about 30% aluminum oxide particles, exhibits high fracture toughness (19 MPa-m^1/2^) and extremely high bending strength (1400 MPa) [199]. In addition, the increased resistance to low-temperature fracture suggests that the material will have long-term reliability. A histologic analysis of cross-sections of implanted Ce-TZP/Al_2_O_3_ ceramic fixators showed not only perfect biocompatibility but also a high rate of bone integration [200]. For example, the bone-to-implant contact (BIC) rate after eight weeks was 80%, which was comparable to the BIC rates with cp-Ti (85%) and Ti-Zr (72%) implants and much higher than those for ZrO_2_ (22–48% [216]) and Ti-6Al-4V (30%) implants. In addition, Ce-TZP/Al_2_O_3_ implants adhered tightly to the surrounding soft tissues with no signs of inflammation [200].

The wide implementation of ceramic implants is hindered by the relatively low machinability of ZrO_2_ [217,218], increased brittleness of ceramics [219,220], low thermal conductivity and sensitivity to thermal shock [206,221,222,223,224,225], and CAD/CAM technologies of mechanical processing of products, which have led to the increase in the volume of studies, the object of which are implants from ceramic materials (for the last 20 years, the number of publications with studies on zirconium implant properties has increased 2.5 times; see Figure 6), with the market for dental implants made of zirconium dioxide increasing by more than 12% per year [226].

### 2.3. Tantalum and Its Alloys

Tantalum, like titanium, is a strong and bioinert metal. However, it is used much less frequently because it is a rare-earth metal (titanium is found in the Earth’s crust almost 2500 times more often than tantalum [227,228]). Moreover, tantalum is less strong but more ductile (1.4 times) and refractory (1.8 times) than titanium. At the same time, the complexity of the production of metallic tantalum from ore containing hundredths of a percent of (Ta,Nb)_2_O_5_ and then the use of expensive processes for manufacturing tantalum products (vacuum-arc, plasma melting, or powder metallurgy methods) [228] eventually sharply increase the cost of the material.

Compared to titanium, Ta has higher thermal and electrical conductivity [227,228] and high corrosion resistance [229] and has been shown to have better biocompatibility (the Ta surface promotes the better adhesion, proliferation, and differentiation of osteoblasts compared to the titanium alloy Ti-6Al-4V) [230,231]. In addition, tantalum (even coated on a titanium substrate) shows good antibacterial activity against *Streptococcus mutans* and *Porphyromonas gingivalis* [232].

Basically, tantalum implants are made of highly porous “pure” Ta, the so-called Trabecular Metal™ (TM) [30,233,234,235,236]. Its open-cell structure has interconnected pores, as a result of which the structure becomes highly porous (75–80%) and resistant to fatigue failure and retains its strength throughout the healing process [237]. Such tantalum implants are capable of osteointegration and biological fixation with the growth of new bone tissue in the pores and trabeculae (small cells or labyrinths, which resemble the own trabeculae of the bone substance) of the implants, and no fibrotic changes are detected at the “bone–implant” boundary [238].

The modulus of elasticity of porous Ta (1.3–10 GPa [229]) is similar to the modulus of elasticity of subchondral bone [237,239] and more similar to that of natural cortical (12–18 GPa [238]) and spongy bone (0.1–0.5 GPa [229]) in comparison with traditional cp-Ti and titanium alloys (106–115 GPa [94,229]), which contributes to stress reduction and the preservation of bone stock. Simultaneously, the high coefficient of friction allows TM to demonstrate higher biomechanical stability compared to conventional cementless implants even in conditions of bone deficiency or insufficient bone density [240].

However, the creation of a material with a highly porous structure leads to a decrease in its mechanical properties [241], which can limit its use in load-bearing structures, while the decrease in the strength of the implant material can impair its mechanical stability in vivo and increase the frequency of implant failures [108]. Thus, the compressive strength of highly porous Ta is 60 MPa (which is significantly higher than the values of compressive strength, though, compared to porous (75%) titanium (~25 MPa) [242]), the tensile strength is 63 MPa, and the bending strength is 110 MPa; in a study, the compressive fatigue endurance limit was 23 MPa at 5–106 cycles, and the samples showed significant plastic deformation [243]. On the other hand, tantalum implants with low porosity may not provide the necessary surface area for cell attachment and tissue ingrowth [219]. Also, the efficiency of bone tissue “sprouting” into the pores of tantalum implants is influenced by the pore size. A histological analysis of highly porous tantalum specimens showed a significant increase in the degree of filling of the implant pores with bone tissue with time. The authors [237] noted that implants with a smaller pore diameter (430 microns) have better mechanical properties in comparison with more “porous” (650 microns) implants. The optimal pore size for promoting bone ingrowth in metallic porous materials, as previously mentioned, is 100–500 μm [127,219], providing a large surface area for cell attachment and proliferation, which can promote both new bone formation as well as the diffusion of nutrients and waste products, which is important for promoting cell survival and proliferation [219].

However, tantalum has a number of disadvantages, such as the high cost of the material, and there are difficulties in manufacturing the precise internal relief of the implants, which also increases the cost of tantalum implants, so they are not available to all patients. As a consequence, the dental implant can be made in a hybrid design (titanium alloy anchoring base with a trabecular tantalum middle part) [30,234,244,245] and also form tantalum coatings on implants made of other materials [126,246,247,248,249,250].

In addition, search studies are underway to develop biocompatible alloys of increased strength based on tantalum, in particular Ta-Ti [108,162] or bioactive Ta-Cu alloys [251].

The influence of tantalum content on the properties of binary Ti-xTa alloys (where x = 10–80 wt.%) has been discussed in detail above. Let us only note that the Young’s modulus and tensile strength values of Ta-Ti alloys with tantalum contents of 50 to 80% are in the ranges of 67–102 GPa and 530–685 MPa, respectively [162] (for comparison, the tensile strength of pure compact Ta is 206 MPa [228]). At the same time, the maximum strength has been shown by the Ti-60Ta alloy, and the minimum elastic modulus has been shown by the Ti-70Ta alloy.

The addition of copper to tantalum leads to an increase in its antibacterial properties but can cause minor cytotoxicity and reduce the corrosion resistance of Ta [251]. Thus, the sintered Ta-5Cu alloy shows increased antibacterial activity against *Escherichia coli (E. coli)* due to the prolonged release of Cu ions. At the same time, tribological tests in pin-on-disk sliding geometry on stainless steel (pin-on-disk wear tests) have revealed a much lower coefficient of friction but higher wear rate for the Ta-5Cu alloy compared to pure tantalum [251]. This can be explained by the fact that softer and more ductile copper acts as a “hard lubricant”. The increased wear rate of the Ta-5Cu alloy is also consistent with the data on the decrease in its hardness compared to Ta (3.6 GPa vs. 4 GPa) [251].

Thus, tantalum and its alloys are promising materials for application in dental implantology, but additional research is required for highly porous structures of alloyed tantalum alloys, which give an opportunity to combine high strength with a low modulus of elasticity.

### 2.4. Other Materials for Dental Implants

A number of other materials are also known that are capable of forming a strong bond with bone tissue.

As previously stated, some of the first materials used for implant fabrication were stainless steels, particularly the austenitic steels AISI 316 and 316L [252,253,254] and the ferritic steels AISI 444 and NeoM (the steel of commercially marketed dental implants) [255]. However, the most recent studies where steel implants were the subject date from the 2010s; in the 2020s, steel implants have only been mentioned in review articles or used for control groups (for comparison). Although stainless steels are a stronger and mechanically reliable class of metal alloys, their use as biomaterials is limited due to their lack of proper biocompatibility and tendency to corrode over time in biological environments [219].

As a result, modern dental implant manufacturers have abandoned steel implants because steels have the highest modulus of elasticity (~210 GPa [209]), the lowest corrosion resistance in biological media [254], and the lowest biocompatibility [219,253]. Thus, comparative in vitro tests of the corrosion resistance of commercially available mini-implants made of stainless steel AISI 316 and titanium alloys Ti-6Al-4V (Grade 5 and Grade 23 with reduced oxygen content), when immersed in artificial saliva, saliva with probiotic bacteria *Lactobacillus reuteri,* and saliva with an oral antiseptic—chlorhexidine (CHX)—revealed an increase in the roughness of steel implants when exposed to CHX, as well as a decrease in their microhardness compared to unexposed implants when exposed to CHX and probiotics [254]. It is worth noting that exposure to probiotics significantly increased the roughness of class 5 titanium implants compared to other media, but the microhardness of the samples did not change after exposure to either medium. At the same time, the corrosion resistance of austenitic steels can be increased by applying appropriate heat treatment (by correctly selecting the temperatures of the hardening and subsequent aging of the steel) [252].

In addition, stainless steel AISI 316L demonstrates low integration in contact with the surrounding bone tissue, and the duration of implant healing can reach several months [253].

The osseointegration of steel implants has been attempted to be improved by fabricating products using additive manufacturing techniques for dental implants without the use of subsequent surface treatment [256]. The samples in one study had higher roughness and lower surface energy than commercially realized implants.

The ferromagnetic properties of ferritic stainless steels make them potential materials for use as magnetically bonded implantable dental devices [255]. Comparative studies on the corrosion resistance and cytotoxicity of ferritic stainless steel AISI 444 (ferritic steel alloyed with Nb and Ti) have been carried out, and NeoM steel (ferritic steel for commercialized dental implants) and austenitic steel (the composition of which corresponds to ISO 5832-1, [257]) have revealed an increased tendency towards pitting corrosion in phosphate buffer solution (PBS). The resistance to pitting corrosion of AISI 444 steel is comparable to that of austenitic steel [255].

Taking into account the biocompatibility and high chemical resistance of ceramic materials, there have been studies suggesting Al_2_O_3_- [258,259] and Si_3_N_4_ [24,260,261]-based ceramics as promising biomedical materials for the production of dental implants. Table 2 presents the mechanical properties of ceramic materials used for the production of dental implants in comparison with traditional titanium alloys.

Aluminum oxide (Al_2_O_3_) has high hardness and wear resistance, hydrophilicity, corrosion resistance, good thermal conductivity, and a low coefficient of friction, but has low bending strength [274]. It has been shown [258] that the surface wettability of Al_2_O_3_ and Y-TZP samples is within the hydrophilic regions (the contact angle for Al_2_O_3_ was ~64°, and for Y-TZP, it was ~58°; for comparison, Ti-6Al-4V alloy has a high wetting angle of ~85° [262]). This accounts for the better cellular adhesion of ceramic materials (approximately similar for both types of ceramics) compared to titanium alloy. Meanwhile, the cell adhesion of human osteoblasts (HOB), human osteoblast-like cells (MG-63), and human mesenchymal stromal cells (hMSC), as well as cell spreading and the number of focal cell contact points, were further increased via the covalent immobilization of alkaline phosphatase (ALP), an enzyme involved in bone mineralization, to the surfaces of ceramics [258].

In recent years, silicon nitride (Si_3_N_4_) has attracted increasing attention. Nitride ceramics have increased hardness and strength compared to other types of ceramics (see Table 2), also showing excellent biocompatibility [24,260,261]. It was found that when Si_3_N_4_ samples were immersed in SBF solution, the pH and ionic conductivity values of the samples varied widely in the first days, but then stabilized around SBF values (pH = 7.26; C = 9.148 mS/sm) afterwards (5–6 days). The exposure of silicon nitride ceramics to the MG-63 cell line for 24 h results in low lactate dehydrogenase (LDH) activity and, therefore, a good percentage of cell viability. In addition, the cells grow and proliferate near the samples under normal conditions [261].

Also, nitride ceramics showed good osseointegration in a study. For example, three months after surgery, histologic sections showed superior new bone formation around the tested Si_3_N_4_, polyether-ether-ether-ketone (PEEK), and cp-Ti implants compared to Ti and PEEK (69%, 24%, and 36%, respectively) [275].

At the same time, the strength of Si_3_N_4_ depends on porosity, which, in turn, depends on the sample fabrication technology—in particular, on the sintering temperature (the sintering pressure and holding time had almost no effect on the strength of the product) [261].

In addition, Si_3_N_4_ exhibits antibacterial properties, which proves a threefold increase in the biofilm inhibition area on Si_3_N_4_ samples compared to cp-Ti samples under the same conditions [261]. This gives an advantage in using nitride ceramics for dental implants over titanium and zirconium alloys—a decisive advantage over available Ti or Zi dental implants, as the antibacterial properties may help cope with the continuous increase in the prevalence of peri-implantitis [260].

As biomaterials for dental implant manufacturing, polymeric materials have also been tried; in particular, polyether-ether-ketone (PEEK) [23,26,195,262,275,276,277,278,279,280] is an X-ray-negative semi-crystalline thermoplastic that combines strength (100–230 MPa depending on the polymer base), hardness, and wear resistance, as well as high chemical resistance and biocompatibility [26]. In addition, unlike metallic and, even more so, ceramic materials, PEEK has an elasticity modulus close to the values of the bone tissue elasticity modulus, and it can be regulated by adding carbon particles or fibers to the PEEK composition (which also leads to an increase in polymer strength) [26,277]. Thus, when short carbon fibers are added, the elastic modulus of PEEK is 4–18 GPa [26].

At the same time, in a study, PEEK samples had better adhesion, viability, and proliferation of osteoblasts and gingival fibroblasts compared to titanium, and had indicators similar to those to ZrO_2_ samples, which correlated with the increased wettability of these materials [195], but in comparison with titanium implants, PEEK is less osteoconductive than titanium (less bone–implant contact is observed than with titanium implants) [278]. PEEK also affects the biofilm structure and reduces the likelihood of inflammation around the implant [23].

Moreover, the osteointegration of PEEK implants can be improved by modifying treatment, increasing the surface hydrophilicity [23,262,276,279,280], or by forming bioactive coatings on the surface, which, according to the authors of [23], enhances cell adhesion, proliferation, biocompatibility, and the osteogenic properties of the polymer. Thus, in a study, sandblasting treatment with Al_2_O_3_ microparticles (110 μm in size) of PEEK implants increased bone ingrowth and the degree of implant contact with bone tissue (51.1% for treated PEEK, 30.9% for untreated PEEK), and the osteointegration of treated PEEK was similar to the osteointegration of the titanium implant, the degree of implant contact with bone tissue of which was 54.0% [276,280]. In another study [279], it was shown that laser-modified titanium and PEEK surfaces led to the directed attachment of gingival fibroblasts.

It has also been shown that the shear strengths at the interface between the bone and implants made of carbon fiber-reinforced PEEK (CFR/PEEK) and cp-Ti with hydroxyapatite (HA) coatings were comparable [277], which can be explained by the fact that in both cases, the same materials (bone and hydroxyapatite) come into contact, and the adhesion of HA with implant materials (titanium and PEEK) is the same [277]. At the same time, HA-coated specimens have better osseointegration in comparison with uncoated specimens (in a study, the shear strength at the interface between bone and implants was 8 MPa for uncoated specimens and 15 MPa for coated specimens) [277].

However, there have been studies that do not recommend the use of unmodified PEEK (without surface modification) for implants [278,281]. PEEK compared to Ti and ZrO_2_ showed the highest values of total strain, demonstrating reduced Mises stresses in implants and abutments, but had high tensile stresses in trabecular bone, reaching the critical values [276]. Additional comparative animal and clinical studies are needed to determine the potential of PEEK as a biomaterial for dental implants.

In addition, there have been studies in which, for the production of dental implants, the prospect of composite material application has been considered, i.e., materials consisting of two or more heterogeneous materials (with essentially different physical and/or chemical properties), with a clear interface between them, combining the properties inherent in several constituent materials—for example, metal–ceramic materials, metal–polymer materials, etc.

In [259], the microstructural and mechanical properties and biocompatibility of the composites “hydroxyapatite–ceramic” HA-Al_2_O_3_ and HA-ZrO_2_, with the addition of 5 and 10 wt.% of commercial inert glass (CIG; contains ~69% SiO_2_, ~17% Na2O, ~9% CaO, and ~2% Al_2_O_3_ and MgO and other oxides [259]), were investigated. With an increasing CIG content, the compressive strength and microhardness of HA-Al_2_O_3_ composites increase while the microhardness of HA-ZrO_2_ composites also increases and the compressive strength decreases. The biocompatibility (in vitro and in vivo) and mechanical properties of HA-Al_2_O_3_ composites (microhardness of HV 43 and compressive strength of 36 MPa at sintering temperature of 1200 °C and 10% CIG content) are lower than those of HA-ZrO_2_ composites (HV 47 and 53 MPa under the same conditions).

One of the most important properties to be considered in the fabrication of all-ceramic dental implants is cyclic fatigue because implants are subjected during mastication processes: precisely, during cyclic loading, which can lead to fatigue cracking (appearance of subcritical cracks) and sudden implant failure (implant fracture). It has been shown [282] that ZrO_2_-Al_2_O_3_ (80:20) composite can have up to 20 years of service if the implant diameter is chosen correctly with regard to cyclic fatigue. At the same time, the hardness of the ZrO_2_-Al_2_O_3_ composite is HV 15.2 GPa, and the tensile strength is 700 MPa, which is twice as high as the strength of some types of ceramics and comparable to the tensile strength of titanium alloys (see Table 2).

To improve tribocorrosion properties, a composite material, (Ti-6Al-4V)-PEEK, was created [283]. Ti-6Al-4V alloy is characterized, as noted above, by high corrosion properties due to the formation of a passive TiO_2_ film on the surface, but, as a result of tribochemical reactions occurring at the interface of the “implant-bone”, it can be destroyed (under the action of both wear and corrosion), which becomes the reason of metal ions released in a toxic concentration (metallosis phenomenon) [283]. In one study, the impregnation of a Ti-6Al-4V alloy with a lattice structure obtained using selective laser melting with polyether-ether-ether-ketone (PEEK) at hot pressing allowed to increase the wear and corrosion resistance of composite (Ti-6Al-4V)-PEEK in comparison with samples from titanium alloys, both dense and cellular structured (the specific wear rate of the composite on tribological tests in phosphate buffer solution decreased by 450% in comparison with the sample from Ti-6Al-4V, cut from a rolled bar, i.e., obtained using the traditional method) [283].

To avoid problems related to both the mobility of the components of a prefabricated dental implant (between the implant and abutment and the abutment and crown) as well as bacterial colonization in the gaps of the mechanical connections, thus preventing the fusion of the gingiva or bone tissue with the implant, which can lead to dental implant rejection [284], a one-piece implant consisting of two layers was developed: a zirconia ceramic was used for the crown and a titanium alloy was used for the implant body. The two-layer monolithic metal–ceramic dental implant (Ti-6Al-4V)-YSZ, fabricated using spark-plasma sintering, showed good mechanical properties and biological properties investigated in vitro (good biocompatibility, non-cytotoxicity, cell adhesion, and hemocompatibility) [25].

Thus, there is a great variety of biomaterials that are promising for use in dental implantology. There is a large amount of clinical and statistical material that confirms the well-known thesis that medicine is the science of alternatives—in this case, the alternative consists of using different materials in different clinical situations [174]. However, unfortunately, to date, there has been no clearly structured methodology for the selection of an implant material for specific conditions of use (specific clinical case). Apart from one recommendation, a comparison of the aesthetic performance and durability of implants made of titanium and its alloys (as the main materials of dental implants) with their ceramic counterparts indicates that for implants of load-bearing structures, more durable metal alloys based on titanium and/or zirconium are preferred for masticatory teeth, and in the smile area, more aesthetic white ceramic materials are preferred (based on stabilized zirconium dioxide or silicon nitride (to a lesser extent, it has a gray shade)); however, it is necessary to take into account the following factors [285].

## 3. Implant Surface Modification

The positive course of the processes of dental implant integration into the bone is characterized by at least three indisputable criteria [286]:

(1) An absence of rejection reactions expressed in the development of inflammation in the adjacent tissues, local necrotic changes, and systemic manifestations such as allergic and immune reactions;
(2) The formation of morphofunctional determinants of the integration process in the area of contact, i.e., the “implant–tissue medium”: bone or bone-like substance (in the case of osteointegration);
(3) The relative stability (including mechanical stability) of the above-mentioned morphofunctional determinants for a certain period of time as a reflection of the dynamic equilibrium occurring in the system, i.e., the “implant–tissue substrate”.

Thus, the main operational properties of dental implants, such as osteointegration and biocompatibility, depend primarily on the properties of their surface layer, as it is the implant surface that makes contact with biological tissues of the body (bone, gingiva).

In this connection, the surface modification of dental implants plays an important role, and the majority of scientific studies are devoted to it. Thus, all methods of surface modification can be divided conditionally into groups (Table 3): mechanical, physical, chemical, biochemical (according to the main influence on the surface), and combined (using several types of processing or influences).

According to the influence of the implants’ surface modification on their functional properties, all the methods can be divided into two large groups:

(1) Methods aimed at changing the surface roughness of implants to improve their integration;
(2) Methods forming protective and/or bioactive coatings on the implants to improve their corrosion resistance, biocompatibility, biomechanical stability, and antibacterial properties and to promote bone tissue regeneration.

The requirements of biomechanical compatibility and the fixation of the implant in the body tissues can be satisfactorily solved if we use materials with rough surfaces to which the living tissues are able to firmly attach. In this case, two ways of connection between the implant and the living tissue are created: mechanical coupling as a result of the tissue formation (sprouting) in the implant pores and chemical coupling due to the interaction of the tissue with the components of the elemental composition of the implant. In a study [372], it was shown that due to the rough surfaces of ADiN Touareg implants, the surrounding tissues firmly attach to them and grow into the pores, and when unscrewing such products, there is a significant traumatization of tissues around the implantation site. Smooth polished 3S implants have a smaller area of contact with body tissues. They are fixed using bicortical implantation, are easy to unscrew, and do not tear the surrounding tissues during removal. Thus, on the removed ADiN Touareg implants, there were many tissue fragments, and on the 3S products with smooth surfaces, there were practically no tissue fragments. This indicates the better osseointegration of the rough implant. At the same time, there were no significant histological differences in the bone tissue after the implants with rough and polished surfaces were added, and no significant differences between the states of the surrounding tissues 2 and 6 months after the implantation of each product.

The most frequently used methods for changing the implant surface microgeometry are anodic oxidation, acid/alkaline etching, a combination of these methods, hydrogen peroxide treatment, the formation of coatings by using different methods (the sol-gel method, chemical deposition, etc.), and the mechanical treatment of the implant surface (sandblasting, machine dashing, and laser ablation) (see Table 3).

Another important factor affecting osseointegration is hydrophilicity/hydrophobicity. Thus, hydrophilic materials with a surface tension higher than 30 dyne/cm interact more closely with biological fluids, cells, and tissues and, consequently, contribute to a better osseointegration process [350,377]. The roughness of the implant surface also plays a certain role in this process. Fibroblasts and epithelial cells adhere more strongly to smooth surfaces, and the ability to ensure osteoblast proliferation and collagen synthesis is more pronounced on a surface with moderate roughness [377].

Recently, surface modification via applying various functional coatings has become widespread. This is mainly aimed at improving the integration of implants (foreign bodies) with the body tissues and fighting against peri-implant infection, which can lead to the rejection of artificial products.

Thus, several new technologies of applying hydroxyapatite (Ca_10_(PO_4_)_6_(OH)_2_), calcium phosphates (CaP), and tricalcium phosphates (Ca_3_(PO_4_)_2_) on the implant surface have been developed [7,277,378,379,380,381,382,383]. It has been proved that CaP compounds promote the formation of direct connections with bone tissue in comparison with implants without the additional coating of the titanium surface.

A separate group consists of bioactive surface modifications of dental implants [384,385,386,387]. Mainly, various osteoinductive growth factors are used [11,375,388]. Moreover, to decrease the risk of infectious complication development, the application of antibacterial preparations on the implant surface [20,389,390] and introduction of metal particles (antibacterial components such as Cu, Zn, Ag, and Ga) into the implant surface or into the coating composition are widely used [16,17,18,19,117,149,163,164,232,251].

The analysis of a large number of controversial results of studies devoted to the contact processes at the “implant–bone” interface allows us to conclude that there is no unambiguous opinion about the “correct” way of modifying the surface of dental implants. The problems of improving the quality of dental implantation and combating the complications of this procedure cannot be solved only by applying other substances on the surfaces of implanted materials. It is necessary to solve the multifactorial problem of increasing the efficiency of dental implantology by choosing the optimal implant material, its manufacturing technology, and the method of surface modification, including the formation of a branched “porous” structure on it and giving it bioactive properties.

## 4. Conclusions

Based on the results of this review, the following conclusions were drawn:

- There is a great variety of biomaterials that are promising for application in dental implantology; however, unfortunately, to date, there has been no clearly structured methodology for implant material selection for specific operating conditions (specific clinical cases) or rational technology of implant manufacturing from the selected material;
- There is a need for a complex approach to improve the quality of dental implants, including the choice of the optimal material, implant manufacturing technology, and the method of its surface modification;
- The modification of the dental implant surface should combine different methods aimed at creating the surface texture and formation of bioactive coatings;
- A comparison of the aesthetic indicators and durability of metal and ceramic implants indicates the following for implants of bearing structures: for masticatory teeth, more durable metal alloys based on titanium and/or zirconium are preferable, and in the smile zone, more aesthetic white ceramic materials are preferable (based on stabilized zirconium dioxide or silicon nitride (to a lesser extent, it has a gray shade));
- Among metal alloys, the most promising are alloys of the Ti-Nb-Zr system alloyed additionally with Ga and/or Sn, but the elasticity modulus of these alloys (dense structure) exceeds the elasticity modulus of bone tissue; to reduce the elasticity modulus and increase the bioactivity and corrosion resistance of metal alloys, it is promising to develop composite meta-materials from these alloys (i.e., manufacturing of alloys of the specified composition of the lattice structure using additive technologies, with further “impregnation” of them with polymeric bioactive materials);
- A promising direction is the creation of biomedical nanomaterials; nanotextured surfaces have a positive effect on bone tissue cells and have an antibacterial effect. Promising in the production of dental implants is the use of nanostructured titanium, the advantage of which is the absence of toxic elements (aluminum and vanadium) and the higher strength and corrosion resistance inherent in unalloyed titanium;
- Due to the development of additive technologies, it has become possible to manufacture products with a “controlled” gradient of properties by volume; a one-piece implant consisting of two layers has been developed: zirconium ceramic, used for the crown, and titanium alloy, used for the body of the implant. This has avoided the problems of both the mobility of the components of a prefabricated dental implant (between the implant and abutment and the abutment and crown) as well as the colonization of bacteria in the gaps of mechanical connections, thus preventing the fusion of the gingiva or bone tissue with the implant, which can lead to the rejection of the dental implant; however, it should be taken into account that the heterogeneity of implant and abutment materials may increase corrosion processes due to galvanic processes, so further studies both in vitro and in vivo are required.

Thus, further clinical and experimental studies are needed to select implant materials, processing methods, and surface modification.

To date, there has been insufficient information on dental materials that have optimal mechanical properties, i.e., high strength and low modulus of elasticity (equal to the modulus of elasticity of bone tissue). According to the existing data, varying and/or modifying the chemical composition of materials has not yet allowed the simultaneous achievement of these parameters. Therefore, one of the promising directions of research in this field can be comparative studies of dental implants made of the same alloy but using different methods (traditional and additive technology methods). These studies will provide new insights into the influence of material architecture (macrostructure, porosity, meta-structure) on its mechanical properties (compressive strength, modulus of elasticity). It is also worthwhile to continue research in the field of composite metal–polymer meta-materials or partially biodegradable materials that initially have, or form in the process of use, “macro-pores” necessary for the better osteointegration of implants.

## Figures and Tables

**Table 1:** Titanium alloys used for the production of dental implants.

Double Alloys	Triple Alloys	More Complex Alloys
Ti-Al	Ti-Al-V [62,63,64,65,66,67]	
	Ti-Al-Nb [77,78,79,80]	Ti-Al-Nb + YSZ (ZrO_2_, stabilised Y) [81]
Ti-Nb [53,82,83,84]	Ti-Nb-Zr (TNZ alloys) [76,85,86,87]	Ti-Nb-Zr-Ta [88,89]
		Ti-Nb-Zr-Ta-Si [90]
	Ti-Nb-Ta [91]	
	Ti-Nb-Cu [92,93]	Ti-Nb-Cu-Ga [93]
	Ti-Nb-Ga [93,94,95]	
	Ti-Nb-Mo [75,96]	Ti-Nb-Mo-Zr-Sn [97]
		Ti-Nb-Mo-Ta [98]
Ti-Zr [68,69,70,71,99,100,101,102,103]	Ti-Zr-Nb [104,105]	Ti-Zr-Nb-Ta [104]
	Ti-Zr-Fe [106]	Ti-Zr-Fe-Si [107]
Ti-Ta [108,109,110,111,112]	Ti-Ta-Zr [113,114]	Ti-Ta-Zr-Nb [115]
		Ti-Ta-Zr-Mo [116]
Ti-Cu [117,118,119]	Ti-Cu-Al [120]	
	Ti-Cu-Nb [121]	
Ti-Mo [122,123,124]	Ti-Mo-Zr [123]	Ti-Mo-Zr-Fe [125]
Ti-Mg [126,127,128,129]	Ti-Mg-Sr [130]	
Ti-Fe [131]	Te-Fe-Ge [132]	
Ti-Ca [133]		
Ti-Pd [133,134,135]		
Ti-Pt [133]		

**Table 2:** Mechanical properties of basic materials used for dental implants [26,93,101,154,161,205,206,219,261,262,263,264,265,266,267,268,269,270,271,272,273].

Material	Compressive Strength, MPa	Tensile Strength, MPa	Modulus of Elasticity, GPa	Hardness HV, GPa
ZrO_2_	1300–2000	150	190–210	10–13
Al_2_O_3_	2100–2500	260	221–400	12.5–21
Si_3_N_4_	2200–3000	<350	210–300	15–22
PEEK	118–240	100–230	3.6–3.8	0.7
PEEK with carbon fibres	280–300	250–260	14–18	0.8
cp-Ti	235–353	860	100–105	1.8–2.2
Ti-6Al-4V	990–1565	689	110–115	3.1–3.5

**Table 3:** Methods of surface modification of dental implants.

Group	Method	Features (Characteristics) of the Method
Mechanical treatment	Machining through cutting (machine stroking) [7,287,288,289,290,291,292]	It is performed in order to increase the surface roughness in order to increase the osteointegration of the implant. This method is inefficient, as the rough surface obtained by this method is much inferior to the porous one as per this indicator. The main materials of implants belong to the class of hard-to-machine materials.
	Sandblasting [7,293,294,295,296,297]	A simple and inexpensive method. Cell adhesion, proliferation, and the differentiation of osteoblasts are improved. However, it is often necessary to carry out acid etching of the implant to homogenize the surface microprofile in order to remove the remaining sand particles. Otherwise, the inhomogeneity of the surface material reduces the corrosion resistance of the implant.
Physical Techniques	Additive technologies [25,285,298,299,300,301,302,303,304,305,306,307]	It allows to create complex three-dimensional structures (e.g., trabecular, heroic), which increase the osteoconductivity of implants. Compared to traditional methods of manufacturing complex profile products, it is possible to save resources (materials, energy, labor costs).
	Laser ablation (including laser surface texturing) [288,290,308,309]	Compared to machining, surface texturing reduces the operating costs of the production process (no consumables (e.g., cutting tools)). Provides an effective way to clean the surface of the product without the use of any chemicals. Ability to automate the texturing process with high precision. Allows the creation of micro-caverns—cavities with a diameter of a few micrometers—with an orderly programmed structure. Increases the osseointegration of the implant due to the creation of the precise “branched” macrostructure (topography) of the surface. Highly energetic and expensive processing method.
	Electron beam (EB) structuring/surface texturing [310]	It differs from laser texturing in that instead of a laser beam, a sharply focused beam of electrons moving at high speed is used for technological purposes. EL texturing shows a statistically significant reduction in bacterial adhesion in the absence of antibacterial agents (even after implant polishing). High-energy processing method.
	Plasma spraying [7,253,311]	Economical and safe method. Possibility of applying a wide variety of metal, ceramic, or plastic coatings at atmospheric pressure. Possibility of obtaining composite bioactive substances—in particular, osseointegration enhancing and coatings (e.g., porous hydroxyapatite reinforced with titanium).
	Vacuum arc coating (cathodic arc deposition; physical coating deposition (PVD)) [292,299,312,313,314,315,316,317]	The formation of coatings is carried out in vacuum. It is used for the deposition of metal, ceramic, and composite coatings on substrates made of various materials (including low-heat-resistant (up to 200 °C)). To improve the quality of coatings, equipment and technologies are used for the filtration of the microparticle phase from the plasma stream, ion bombardment and etching of the surface are used to clean and thermoactivate it, and ion implantation is used to increase adhesion and modify the outer layer of the substrate [a number of our publications]. By varying both the composition of cathodes and reaction gases as well as coating modes, it is possible to obtain multilayer functional coatings, thereby improving the operational properties of products—in particular, increasing the corrosion resistance of implants and giving the product antibacterial properties.
	Magnetron sputtering method [318,319,320]	High speed of coating formation at low vacuum of 0.1–1 Pa; no overheating of the substrate. Magnetron sputtering can be carried out both on direct and alternating currents. It is used for the formation of protective coatings in order to increase the corrosion resistance and osteointegration of implants. The coatings formed on the implants are, as a rule, single-layer and simple in composition films (oxide, carbide, nitride, or metallic). Complex coatings (e.g., calcium phosphate) can be deposited wherein the composition of each coating corresponds to the composition of the target atomized in argon. It is possible to combine the deposition of coatings with preliminary plasma-immersion ion implantation. The coatings are characterized by high uniformity, relatively low porosity, and high adhesion to the substrate. The compound formed after the chemical reaction is deposited not only on the substrate but also on the entire surface of the chamber (including the sputtering target, which reduces the efficiency and productivity of the process)
	Glow discharge plasma treatment [321,322,323]	It allows to reduce the roughness of the implant surface while increasing its wettability, which contributes to a reduction in bacterial adhesion. Surface treatment using the glow discharge plasma is carried out in vacuum, which makes it possible to combine this operation with the application of the functional coating in the working chamber of the device. By adjusting the plasma composition, it is possible to change the chemical composition of the surface, which leads to changes in the physical and mechanical properties of the implant (in particular, the hardness of the surface and the breaking strength of the “implant-bone” connection).
	Ultraviolet light treatment (UV photofunctionalization) [324,325]	Allows to restore the original surface qualities of “aged” implants and extend their service life by exposing them to ultraviolet light just before insertion into the bone.
	Ion implantation [326,327,328,329,330]	The introduction of functional metal ions (for example, bioactive ones: Ag, Zn) into the surface is carried out in vacuum. This operation can be performed as an independent method of surface modification (for example, to increase osteogenic activity and antibacterial activity of implants), or it can be combined with other technological operations (for example, to increase adhesion of vacuum–arc coatings).
Chemical Techniques	Chemical (including electrochemical) etching: - acid etching (Acid etching) [331,332,333]. - Alkaline etching (Alkali treatment) [289,334,335,336]	It is possible to obtain different surface textures (its roughness) by adjusting the composition of etching solutions (as a rule, acid concentration), temperature, and etching time. Acid etching significantly increases the roughness of the implant surface without changing its marginal wetting angle. Alkaline etching can increase the hydrophilicity of the dental implant surface. Acid etching is performed at a lower temperature and in a shorter period of time than the commonly used alkali etching treatment.
	Electrochemical deposition of coatings, including oxidation (anodizing) [7,292,294,306,337,338,339]	A relatively simple inexpensive method of forming coatings (usually oxide films). Formed coatings have high porosity, which contributes to the improved osteogenesis of implants. Allows to obtain a hydrophilic surface, which contributes to the better osteointegration of implants. It is practically impossible to control the structure and properties of coatings in the process of their deposition. Due to the high level of environmental contamination and danger for personnel, it requires the use of complex treatment and protection facilities.
	Electrophoretic deposition of coatings [340,341,342]	A relatively simple low-temperature method for forming coatings on an electrically conductive substrate. Virtually indifferent to the shape of the coated surface, it has high productivity and is well adapted to mass production. It is possible to control the thickness and morphology of the coating by varying the deposition voltage and time. Possibility of producing polymer, metal–polymer, ceramic, and composite bioactive coatings (e.g., hydroxyapatite coatings with silver and lignin or chitosan coatings).
	Sol-gel method [343,344,345,346]	The synthesis process is relatively simple and allows excellent control over the composition of coatings and their application to surfaces of virtually any shape. Allows to achieve uniform distribution of elements of multicomponent systems on the surface of various solids. Enables the localized delivery of a wide range of drugs (in particular, antimicrobials) at a controlled rate.
	Chemical Deposition of Coatings (CDC) (CVD) [347,348,349]	The high-temperature process of formation of high-purity homogeneous solid films by means of a chemical reaction. Possibility of coating complex implants (including internal cavities of products). Coating is only possible on heat-resistant substrates.
Combined Processing Techniques	Sandblasting of the surface followed by acid etching [70,309,350,351,352]	Combines the advantages of both types of surface modification. - The SLA (Sand-blasted, Large-gri, Acidetched, translated as “sand-blasted; coarse-grained; acid etched”) surface has a coarse-grained structure; implants show better osseointegration and osteoconductivity, especially in the initial phase (after insertion), compared to the untreated surface. - The RBM (Resorbable Blast Media, translated as “resorbable; jet; medium”) surface is formed by sandblasting the implant surface with particles of resorbable Ca-phosphate compound followed by etching in organic low-concentrated acid. It has deeper micropores compared to SLA, which contributes to better osteoconductivity. In addition, it helps reduce osteoporosis. However, the surface after combined treatment is hydrophobic, which, in general, somewhat complicates the osteointegration of implants.
	Plasma electrolytic treatment (e.g., Plasma electrolytic oxidation) [177,302,353,354,355,356,357,358,359,360]	Simple, inexpensive, and environmentally safe method. It allows to apply various (in particular, polymeric) coatings on metal and ceramic substrates with their simultaneous cleaning in order to increase the corrosion resistance of implants. It is possible to realize the microalloying of both the implant surface as well as of the formed coating (for example, the formation of Ca-P coatings with silver nanoparticles)
	Combined treatment with different PVD methods [361]	Allows for the formation of multilayer coatings, each layer of which is deposited in a layer-by-layer manner, such as in the deposition of a multilayer coating comprising an oxide sub-layer obtained via gas-thermal oxidation and a calcium phosphate layer obtained via magnetron sputtering.
	Cold atmospheric plasma assisted vapor phase CVD method (PECVD) [362,363,364,365]	The CVD process uses plasma to decompose the initial substances, activate the substrate surface, and achieve ion etching. Compared to the traditional method, it is low-temperature (due to plasma amplification), so it allows to obtain coatings on non-thermal substrates. Formed oxide coatings have superhydrophilic surfaces. Possibility to apply thin (up to 0.5 µm) glass–ceramic coatings.
	Laser chemical vapor deposition (LCVD) [366,367]	CVD process that utilizes a laser source to decompose starting substances and activate the substrate surface. Allows to obtain glass–ceramic coatings on heat-resistant substrates. By adjusting the laser power and pressure in the chamber, it is possible to obtain ceramic-like oxide films with different microstructures.
Biochemical Techniques	Stem cell culturing [368,369,370]	Stem cells (mesenchymal (MSC), adipose-derived, bone marrow-derived, or embryonic (ESC)) can be successfully grown in culture media (e.g., serum-free and xenogeneic for adipose-derived MSC) for further culturing on various dental implant surfaces to enhance implant osseintegration and bone regeneration.
	Protein-containing coating on implant surfaces [11,371,372,373,374,375,376]	It is possible to incorporate protein (e.g., bone morphogenetic protein (BMP-2) or extracellular matrix (ECM)) into polymeric biodegradable coatings applied to implants. It is possible to apply a liquid protein coating (e.g., platelet- or leukocyte-rich fibrin) to implant surfaces. Used to accelerate healing of soft and hard tissues of the body.

## Data Availability

Data are contained within the article.
